# *CHD7* Mutational Analysis and Clinical Considerations for Auditory Rehabilitation in Deaf Patients with CHARGE Syndrome

**DOI:** 10.1371/journal.pone.0024511

**Published:** 2011-09-13

**Authors:** Mee Hyun Song, Hyun-Ju Cho, Hee Keun Lee, Tae Jun Kwon, Won-Sang Lee, Sanghee Oh, Jinwoong Bok, Jae Young Choi, Un-Kyung Kim

**Affiliations:** 1 Department of Otorhinolaryngology, Kwandong University College of Medicine, Goyang, South Korea; 2 Department of Biology, College of Natural Sciences, Kyungpook National University, Daegu, South Korea; 3 Department of Otorhinolaryngology, Yonsei University College of Medicine, Seoul, South Korea; 4 Department of Anatomy, BK 21 Project for Medical Science, Yonsei University College of Medicine, Seoul, South Korea; Instituto de Ciencia de Materiales de Madrid - Instituto de Biomedicina de Valencia, Spain

## Abstract

**Background:**

Otologic manifestations are one of the most consistent findings of CHARGE syndrome found in more than 90%. Since genetic analysis of the *CHD7* gene has rarely been performed in previous reports dealing with ear abnormalities, the genotypic spectrum of *CHD7* mutations was analyzed in deaf patients with CHARGE syndrome, and the clinical considerations concerning auditory rehabilitation were investigated.

**Methods:**

Nine Korean patients with CHARGE syndrome showing profound hearing loss and semicircular canal aplasia were included. All 38 exons of *CHD7* were analyzed by direct sequencing. For splice site variations, *in silico* and exon-trapping analyses were performed to verify the pathogenicity of nucleotide variations. Clinical features and the outcome of auditory rehabilitation were also analyzed.

**Results:**

Eight of 9 patients revealed alterations of the *CHD7* gene including 3 frameshift, 2 nonsense, 2 splice site, and 1 missense mutations. Five of 9 patients were clinically diagnosed as atypical CHARGE syndrome but demonstrated various mutations of the *CHD7* gene. One familial case showed intra-familial variability. Radiologic findings suggesting cochleovestibular nerve deficiency were identified in most of the patients. Of the 8 patients who underwent cochlear implantation, 5 patients demonstrated favorable outcome. Larger diameter of the cochleovestibular nerve on imaging and absence of severe mental retardation were factors related to better outcome after cochlear implantation rather than the type of *CHD7* mutations. Auditory brainstem implantation was performed in two patients who did not benefit from cochlear implantation.

**Conclusions:**

Genetic analysis of the *CHD7* gene should be performed in cases with semicircular canal aplasia even when other typical features of CHARGE syndrome are absent. For auditory rehabilitation in CHARGE syndrome, cochlear implantation should be strongly recommended in selected cases with favorable prognostic factors. Auditory brainstem implantation may be a viable option in patients with CHARGE syndrome who have failed to benefit from cochlear implantation.

## Introduction

CHARGE is an acronym describing a set of conditions including “C”oloboma of eye, “H”eart malformations, “A”tresia of choanae, “R”etardation in growth and development, “G”enital hypoplasia, and “E”ar anomalies. The combination of these anomalies was first reported by Hall and Hittner [1], [2] in 1979 after which the acronym was proposed by Pagon *et al.* [3] in 1981. The diagnostic criteria proposed by Blake *et al.* [4] in 1998 and those updated by Verloes [5] emphasizing the importance of semicircular canal hypoplasia or aplasia are widely used today for the clinical diagnosis of CHARGE syndrome. The incidence of CHARGE syndrome is approximately 1/10,000 and most of the cases are sporadic although 17 familial cases have been reported to date [6]. The mode of inheritance is autosomal dominance with variable penetrance [6].

Haploinsufficiency of the *CHD7* gene has been identified as the molecular basis of CHARGE syndrome by Vissers *et al.* [7] in 2004. The *CHD7* gene located on chromosome 8q12.1 is 188 kb in size and consists of 37 coding and one non-coding exons [7]. The CHD7 protein comprised of 2,997 amino acids belongs to the chromodomain helicase DNA binding family, one of the four major families of ATP-dependent chromatin remodeling proteins. Approximately 60–70% of patients clinically diagnosed as CHARGE syndrome were found to have pathogenic mutations in the *CHD7* gene [8]. Truncating mutations including nonsense or frameshift mutations are the most frequently encountered forms of genetic alterations followed by splice site or missense mutations found in lower incidences [8]. Chromosomal abnormalities with or without involvement of the *CHD7* gene also have been reported to cause phenotypic features of CHARGE syndrome [9].

Among the various clinical manifestations of CHARGE syndrome, otologic symptoms and signs are one of the most consistent findings which are included as one of the major criteria in both Blake's and Verloes' clinical criteria [4], [5]. Characteristic temporal bone anomalies are reported to be found in 98% of *CHD7* mutation positive cases along with external ear malformations and hearing loss also found in more than 90% [8], [9]. Analysis of temporal bone computed tomography (CT) findings has revealed aplasia or hypoplasia of the semicircular canal, cochlear dysplasia, atresia of bony cochlear nerve canal (BCNC), oval window atresia, ossicular malformations as common characteristics of CHARGE syndrome [10]. Although mild semicircular canal dysplasia is one of the most commonly seen inner ear anomalies, complete aplasia of the semicircular canals with relatively intact cochlear structures is a very rare condition. Since CHARGE syndrome manifests a variety of different conditions that may overlap other syndromes such as velocardiofacial syndrome or Noonan syndrome, aplasia of the semicircular canals may be a decisive clue in suspecting CHARGE syndrome leading to molecular analysis of the *CHD7*gene [11], [12].

Considering that hearing loss of variable degrees is present in most of the cases with CHARGE syndrome, auditory rehabilitation is another important factor in the management of these patients. It has been reported that early auditory rehabilitation is especially important in patients with multiple disabilities for adequate development of communication skills [13]. In CHARGE syndrome patients with severe to profound hearing loss, cochlear implantation (CI) has resulted in variable outcomes because of concomitant multiple handicaps and anatomical factors such as cochlear nerve aplasia [14], [15]. Most of the patients with CHARGE syndrome achieve limited auditory benefit and improved attentiveness and responsiveness following CI but open-set speech perception is acquired in very rare cases [14], [16], [17].

Although there have been many reports concerning otologic manifestations and auditory rehabilitation in CHARGE syndrome, molecular analysis of the *CHD7* gene was not performed in most of the reports [14], [16], [17]. Genetic analysis is especially important in CHARGE syndrome considering that this disorder shares many features with other syndromes which may lead to incorrect diagnosis and that some patients with mutations in the *CHD7* gene manifest only mild symptoms as seen in familial cases of CHARGE syndrome [6]. Therefore, in this study, we have performed *CHD7* mutational analysis in Korean patients with CHARGE syndrome presenting with profound hearing loss and typical inner ear malformations in order to broaden the genotypic spectrum of CHARGE syndrome. The clinical features were also analyzed with emphasis on auditory rehabilitation including CI and auditory brainstem implantation. In addition, factors that may influence the outcome of auditory rehabilitation in these patients were further investigated.

## Materials and Methods

### Subjects

Among the patients who were enrolled in the auditory rehabilitation program at Severance Hospital, nine patients with profound sensorineural hearing loss showing semicircular canal aplasia on the temporal bone CT were included in this study. Eight of 9 patients received CI for auditory rehabilitation, and two children underwent subsequent auditory brainstem implantation because no sound perception was possible using a CI. There were 6 males and 3 females; and the ages of the patients at the time of CI ranged from 14 months to 20 years (mean: 6.2 years). One patient aged 15 years was recommended to receive CI but the parents have refused surgery. Combined disabilities and other medical conditions were reviewed and further evaluations including ophthalmologic examination and echocardiography were performed. Written informed consent was obtained from participating individuals, and this study was approved by the Institutional Review Board of the Yonsei University College of Medicine.

### Genetic analysis

From the peripheral blood of 9 subjects, genomic DNA was extracted using a FlexiGene DNA extraction kit (Qiagen, Hilden, Germany). All 38 exons and flanking intronic sequences of *CHD7* were amplified by polymerase chain reaction (PCR) and the quality of the PCR products were examined by electrophoresis on 2% agarose gels. The sequences of the primers and PCR conditions are provided in Table S1. Each fragment was purified and subsequently sequenced using ABI PRISM Big Dye Terminator Cycle Sequencing Kit (V3.1) and an ABI PRISM3130XL DNA analyzer (Applied Biosystems, Foster City, CA, USA). ABI Sequencing Analysis (v.5.0) and Lasergene–SeqMan software were used for the data analysis. For the identified missense variation, the presence of the variant was evaluated in 100 unrelated Korean subjects who showed normal audiograms. For chromosomal analysis, leukocytes from the peripheral blood were cultured and Giemsa staining of the chromosomes of cells arrested in metaphase was performed for karyotyping.

### Exon trapping analysis

For the two splice site variations, *in silico* splice site prediction was analyzed to identify any possible effects of the variation on the native splicing process. Three online applications were used for each variation (FruitFly; www.fruitfly.org/seq_tools/splice.html, NetGene2; www.cbs.dtu.dk/services/NetGene2 and Human Splicing Finder; www.umd.be/SSF) and the significance was indicated by a score.

For *in vitro* splicing assay, mini-gene system vector was constructed, and the basement vector pSPL3 was kindly provided by Dr. Thomas v. O. Hansen. DNA sequences containing one or two exons adjacent to the splice site variations and the ∼300-bp flanking intronic sequences were amplified from the genomic DNA of patients having heterozygous variations and inserted into the pSPL3 vector (Table S2). After vector sequencing to isolate wild type and mutant constructions, the isolated vectors and a mock pSPL3 vector were transfected into HeLa cells using FuGENE HD Transfection Reagent (Roche Diagnostics GmbH, Mannheim, Germany). The transfected cells were lysed 48 hrs after the tranfection and total RNA was extracted using the RNeasy Mini Kit (Qiagen, Hilden, Germany). With 1 µg of RNA, first strand cDNA was synthesized by reverse transcription using oligo-(dT)_16_ primer and High Capacity cDNA Reverse Transciption Kit (Applied Biosystems, Foster City, CA, USA). Using 1 µl of cDNA as template, PCR amplification was performed with pSPL3 vector specific primers SD6 and SA2, and the size of the generated mock, normal, and mutant type PCR fragments were compared on 2% of agarose gels. The sequence of PCR products were confirmed with direct sequencing or vector sequencing after TA cloning to isolate various fragments.

### Diagnostic criteria for CHARGE syndrome

The criteria proposed by Verloes [5] were applied for clinical diagnosis of CHARGE syndrome (Table 1).The three major signs included coloboma, choanal atresia, and semicircular canal hypoplasia and the minor signs included rhombencephalic dysfunction, hypothalamo-hypophyseal dysfunction, abnormal middle or external ear, malformation of mediastinal organs, and mental retardation. CHARGE syndrome was classified into typical, partial/incomplete, or atypical according to the number of major or minor signs present.

**Table 1:** Diagnostic criteria by Verloes.

Criteria	Definition
Major1. Coloboma (iris or choroid, with or without microphthalmia)2. Atresia of choanae3. Hypoplastic semicircular canalsMinor1. Rhombencephalic dysfunction (brainstem dysfunctions, cranial nerve VII to XII palsies and neurosensory deafness)2. Hypothalamo-hypophyseal dysfunction (including GH and gonadotrophin deficiencies)3. Abnormal middle or external ear4. Malformation of mediastinal organs (heart, esophagus)5. Mental retardation	Typical CHARGE3 major signs2/3 major + 2/5 minorPartial/incomplete CHARGE2/3 major + 1/5 minorAtypical CHARGE2/3 major + 0/5 minor1/3 major + 3/5 minor

### Radiologic evaluations

The temporal bone CT scan was performed with a 16 multidetector row CT scanner (Somatom Sensation 16; Siemens, Erlangen, Germany) using a standard temporal bone protocol. Contiguous 0.7-mm scans of the temporal bone were acquired in the axial plane and reformatted coronally with 1.0-mm increments. CT images were performed, digitally stored, and displayed by using the Picture Archiving Communication System (PACS) (Centricity; GE Healthcare, Milwaukee, WI).

Magnetic resonance imaging (MRI) was acquired by using a 3.0-T (Achieva; Philips Medical Systems, Best, the Netherlands) or 1.5-T system (Intera; Philips Medical Systems, Best, the Netherlands) with a six-channel sensitivity encoding (SENSE) head coil. The targeted parasagittal scan perpendicular to the long axis of the internal auditory canal was obtained with T2-weighted three-dimensional (3-D) turbo spin-echo (TSE) sequence with driven equilibrium RF reset pulse (DRIVE), following routine MR sequences with spin-echo T1- and T2-weighted images. The sequence parameters for the T2-weighted 3-D FSE sequence with DRIVE were as follows: repetition time (TR)/echo time (TE) = 1500/200 ms, 256 acquisition/256 reconstruction, 15-cm field of view, 1.5-mm section thickness with a 0.75-mm overlap, number of acquisitions = 2, and the scan time was less than 5 minutes.

### Audiologic tests and evaluation of speech performances

Audiologic tests including pure tone audiometry, auditory brainstem response, otoacoustic emission were performed before undergoing CI. Overall auditory performances were evaluated by categories of auditory performance (CAP) [18]. Speech evaluations were performed preoperatively and 1, 3, 6, 12, 18, 24 months after either CI or auditory brainstem implantation.

## Results

### Mutations of the CHD7 gene

*CHD7* mutations were identified in 8 of 9 patients showing characteristics of CHARGE syndrome (Table 2). Six mutations were found in the coding sequence while two were intronic variations suspected of splice site mutations. The mutations in the coding sequence included 3 frameshift mutations causing truncation of the protein, 2 nonsense mutations, and 1 missense mutation. The missense mutation was not found in 100 unrelated normal controls. The region of the protein containing the missense mutation, p.R2065S, was identified as highly conserved in various vertebrate species (Figure S1). In one family, the sibling of the proband (Patient 1) identified as having a frameshift mutation of the *CHD7* gene demonstrated the same mutation, whereas no mutation was found in their parents. Five of the 8 mutations identified in this study were novel mutations of the *CHD7* gene. None of these novel mutations were listed in the 1000 genome sequencing data. The *CHD7* gene was not mutated in one patient diagnosed as typical CHARGE syndrome. No chromosomal abnormalities were identified in any of the patients.

**Table 2:** Genetic analysis of Korean patients with CHARGE syndrome.

Patient No.	Clinical diagnosis	Mutation (Nucleotide)	Mutation (Protein)	Type	In-vitro splicing assay	In-silico assay	Ref
1	Typical	c.921–922delAG	p.G308AfsX9	Frameshift			(20)
2	Atypical	c.7331T>A	p.L2444X	Nonsense			Novel
3	Atypical	c.6193C>A	p.R2065S	Missense			Novel
4	Atypical	c.6832insC	p.T2278HfsX3	Frameshift			Novel
5	Atypical	c.5210+5G>C		Splice site	Exon 23 skipping	Deterioration of splicing donor site	Novel
6	Typical	c.222delG	p.Q74HfsX9	Frameshift			Novel
7	Typical	c.5405-7G>A		Splice site	Insertion of 5bp at cryptic acceptor site	New cryptic splicing site	(20–21)
8	Typical	-	-	-			
9	Atypical	c.1465C>T	p.Q489X	Nonsense			(20)

### In silico splice site prediction and in vitro splicing assay

The two intronic variations of the *CHD7* gene located adjacent to the splice site were analyzed further by *in silico* splice site prediction programs and *in vitro* splicing assay to assess whether these variants alter the splicing of pre-mRNA. Three different splice site prediction programs were tested and the results are shown in Table S3. One variation (c.5210+5G>C) was predicted as a loss of strength of native splice donor site in all three prediction programs. The other variation (c.5405-7G>A) resulted in appearance of a new cryptic splice site in two of the prediction programs.

Further study with *in vitro* splicing assay using pSPL3 mini-gene system demonstrated alternative splicing more clearly and supported the prediction results. The variation c.5210+5G>C caused exon skipping as a result of splice donor site deterioration and c.5405-7G>A introduced a 5-bp intronic sequence by activation of a cryptic acceptor site (Figures S2, S3).

### Clinical diagnosis of CHARGE syndrome

The diagnostic criteria proposed by Verloes [5] were applied for the diagnosis of CHARGE syndrome (Table 3). Of nine patients, four patients were diagnosed as typical CHARGE syndrome, while five were categorized as atypical CHARGE syndrome. Because of the inclusion criteria of this study, one major (semicircular canal aplasia) and one minor criteria (rhomencephalic dysfunction; sensorineural hearing loss) were met in all patients. Coloboma was identified in three patients at sites including optic disc (Patients 6 and 8) and chorioretina (Patient 7). One of the patients with coloboma (Patient 7) was blind whereas the other two patients demonstrated preserved vision. Malformations of the mediastinal organs were found in 6 of 9 patients. Five had cardiac anomalies; two patients showed patent ductus arteriosis and 3 patients demonstrated subaortic septal hypertrophy with tricuspid and mitral regurgitation, endocardiac cushion defect, and ventricular septal defect, respectively. Foregut duplication requiring surgical resection was identified in one patient (Patient 1). External ear defects including the characteristic cup-shaped deformity of the auricles were shown in three patients, two of whom also exhibited middle ear defects such as oval window atresia and ossicular deformities. Unilateral incomplete facial palsy, one of rhomencephalic dysfunctions, was shown in three patients.

**Table 3:** Clinical diagnosis of CHARGE syndrome.

Criteria	Patient No.								
	1	2	3	4	5	6	7	8	9
**Major**									
Coloboma						+	+	+	
Choanal atresia	+								
Semicircular canal aplasia	+	+	+	+	+	+	+	+	+
**Minor**									
Rhomencephalic dysfunction	+	+	+	+	+	+	+	+	+
Hypothalamohypophyseal dysfunction	+		+	+	+	+	+	+	+
Abnormal middle/external ear	+	+		+		+			+
Malformation of mediastinal organs	+	+		+	+	+	+		
Mental retardation	+		+		+	+	+	+	
**Others**									
Urogenital anomalies								+	+
Cleft palate/lip				+					
Limb abnormalities									+
Facial dysmorphia						+			
CHARGE syndrome (Verloes)	Typ	Atyp	Atyp	Atyp	Atyp	Typ	Typ	Typ	Atyp

According to the criteria proposed by Verloes [5], hypothalamohypophyseal dysfunction which corresponds to the previous “R” or “G” of CHARGE acronym can be diagnosed only when growth hormone or gonadotrophin deficiencies are demonstrated. Since not all of the patients in this study performed endocrinological laboratory tests, hypothalamohypophyseal dysfunction was considered to be present when definite retardation of development was demonstrated on objective developmental tests. However, genital abnormalities were not included as hypothalamohypophyseal dysfunction in this study to eliminate the male preference in the diagnosis of CHARGE syndrome.

**Figure 1:**
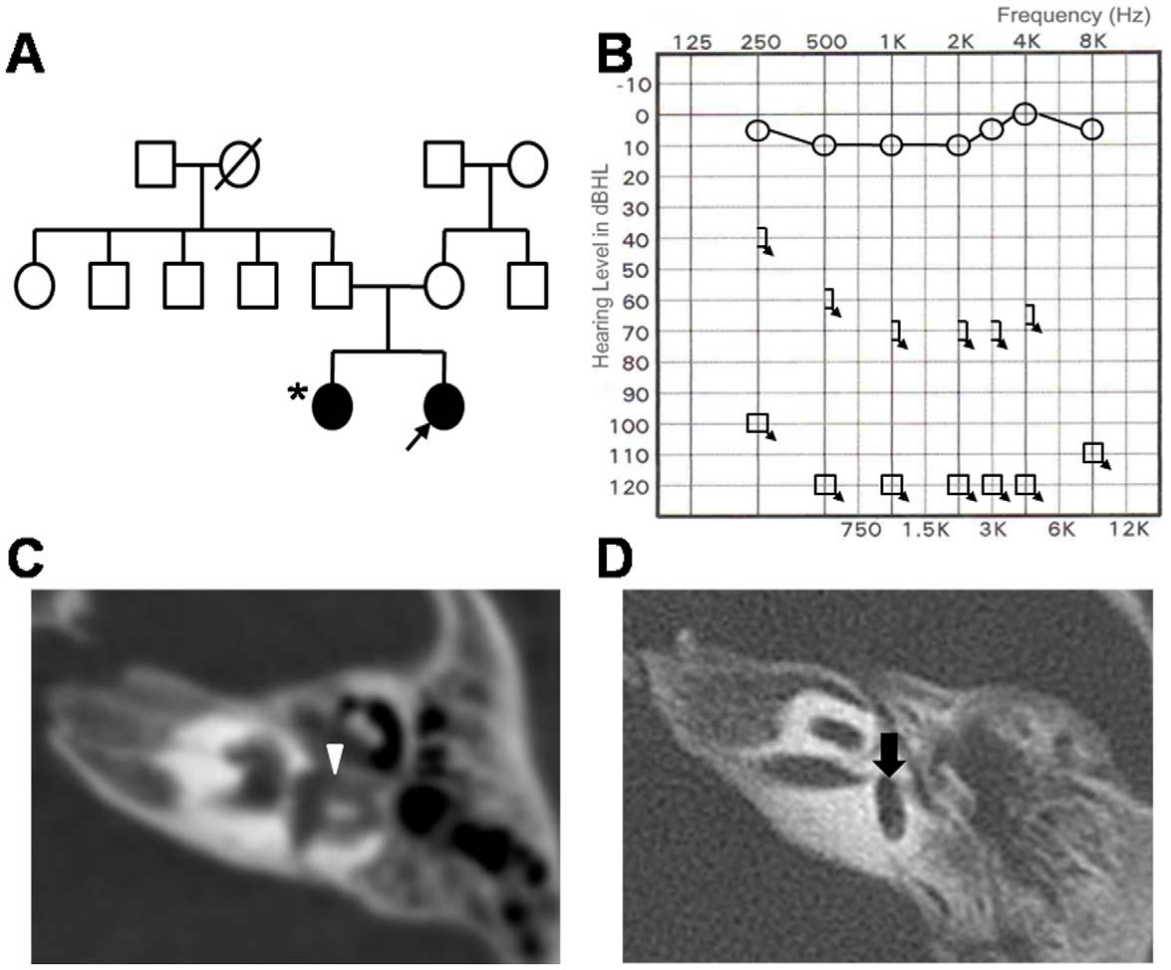
Pedigree and clinical findings of familial case of CHARGE syndrome (Patient 1). (A) Pedigree shows that only two siblings are affected by deafness either bilaterally (Patient 1; proband marked by black arrow) or unilaterally (sister of Patient 1 marked by asterisk). (B) The sister of Patient 1 demonstrated unilateral deafness on pure tone audiometry. (C) Temporal bone CT of the sister of Patient 1 with unilateral deafness showed slightly dilated but present semicircular canals (white arrowhead) with mild cochlear abnormality and normal sized internal auditory canal on the affected side. (D) Temporal bone CT of Patient 1 with bilateral congenital deafness demonstrated complete absence of all semicircular canals (black arrow).

In the familial case (Patient 1), the proband was diagnosed as typical CHARGE syndrome demonstrating features such as choanal atresia, laryngomalacia, foregut duplication, and myasthenia gravis in addition to the sensorineural hearing loss and inner ear malformations. The parents who did not carry any detectable mutations of the *CHD7* gene showed normal hearing on pure tone audiometry (data not shown) without any clinical features of CHARGE syndrome, and the other family members also did not have any subjective hearing loss although they were not audiologically tested (Fig. 1A). The sibling who carried the same frameshift mutation as the proband revealed unilateral profound sensorineural hearing loss and normal hearing on the contralateral side (Fig. 1B). On temporal bone CT, the sibling of Patient 1 revealed slightly dilated but present semicircular canals with slightly hypoplastic cochlea and normal sized internal auditory canals on the affected side, and normal inner ear structures on the contralateral side with normal hearing (Fig. 1C). In contrast, Patient 1 with bilateral congenital deafness demonstrated complete absence of all semicircular canals as well as hypoplastic cochlea and narrow internal auditory canals bilaterally (Fig. 1D). Atrial septal defect was present in the sibling, whereas no cardiac defect was found in Patient 1.

### Radiologic findings

**Figure 2:**
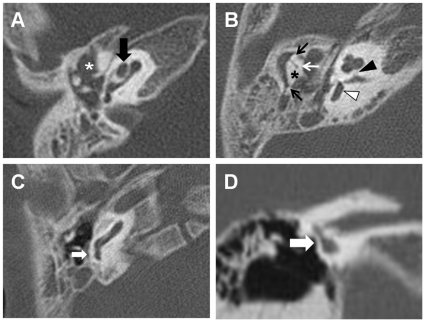
Computed tomography findings of inner ear anomalies typically seen in patients with CHARGE syndrome. (A) Cochlear hypoplasia is shown by a black arrow (Patient 1). (B) Bony cochlear nerve canal is obliterated (black arrowhead) and complete aplasia of the semicircular canals is seen (white arrowhead). The incus is dysmorphic and slightly rotated state (asterisk). Ankylosis between the incus and malleus (white arrow) and between the ossicles and epitympanic bone (black arrows) is shown (Patient 4). (C) Bony obliteration of the round window is seen (small white arrow) (Patient 6). (D) Oval window atresia is seen on the coronal image (large white arrow) (Patient 2).

Temporal bone CT of the patients revealed typical findings of the cochlea and vestibule (Table 4). Complete aplasia of all three semicircular canals and cochlear anomalies consistent with cochlear hypoplasia were identified in all patients (Fig. 2A, 2B). All but one patient exhibited narrow internal auditory canals, and the bony cochlear nerve canal (BCNC) was obliterated in 7 patients (Fig. 2B). Deformities of the incus and stapes were identified in 6 patients, and most of these patients had combined oval window atresia (Fig. 2B, 2D). Complete bony obliteration of the round window was shown in only one patient (Fig. 2C).

**Table 4:** Radiologic findings of patients with CHARGE syndrome.

Patient No.	Age* (Y;M)	Sex	Cochlea	Vestibule	Middle ear	Facial canal	IAC	BCNC	Nerve component on MRI
1	3	F	Cochlear hypoplasia	SCC aplasia	ME opacification, incus/stapes deformity	-	Narrow	Patent	CVN>FN
2	20;8	M	Cochlear hypoplasia	SCC aplasia	OW atresia, incus/stapes deformity	LS, TS	Narrow	Obliterated	CVN = FN
3	4;7	M	Cochlear hypoplasia	SCC aplasia	-	LS	Narrow	Patent	FN>CVN
4	2;5	M	Cochlear hypoplasia	SCC aplasia	ME opacification, incus/stapes deformity, ankylosis, OW atresia??	-	Narrow	Obliterated	FN>CVN
5	2;4	M	Cochlear hypoplasia	SCC aplasia	-	-	Normal	Obliterated	NA
6	1;2	F	Cochlear hypoplasia	SCC aplasia	OW atresia, RW atresia, incus/stapes deformity	LS	Narrow	Obliterated	FN only
7	2	F	Cochlear hypoplasia	SCC aplasia	-	LS	Narrow	Obliterated	FN only
8	4;7	M	Cochlear hypoplasia	SCC aplasia	OW atresia, incus/stapes deformity	LS	Narrow	Obliterated	FN>CVN
9	15**	M	Cochlear hypoplasia	SCC aplasia	OW atresia, incus/stapes deformity	LS	Narrow	Obliterated	NA

*Age at cochlear implantation;

**age at initial visit; IAC: internal auditory canal; BCNC: bony cochlear nerve canal; IP-II: incomplete partition type II; SCC: semicircular canal, ME: middle ear, CVN: cochleovestibular nerve, FN: facial nerve; OW: oval window, RW: round window, LS: labyrinthine segment, TS: tympanic segment; NA; not available.

**Figure 3:**
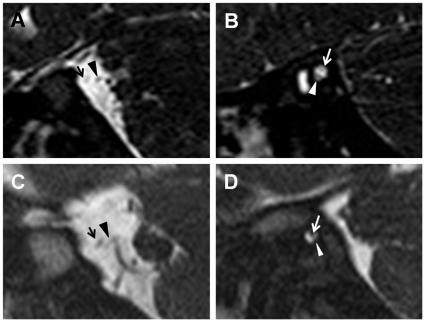
The parasagittal images of temporal MRI in patients with CHARGE syndrome. (A–B) The cochleovestibular nerve (arrowheads) is larger in diameter than the facial nerve (arrows) at the cerebellopontine angle (A) and within the internal auditory canal (B) in Patient 1. (C–D) The cochleovestibular nerve (arrowheads) is smaller in diameter than the facial nerve (arrows) at the cerebellopontine angle (C) and within the internal auditory canal (D) in Patient 4.

On parasagittal view of temporal MRI, the cochleovestibular nerve (CVN) was identified at the cerebellopontine angle and within the internal auditory canal in 5 of 7 patients whose images were available for review (Table 4). Because of narrow internal auditory canals, individual branching of the CVN into the cochlear nerve and superior/inferior vestibular nerves that is usually identified on parasagittal images of MRI could not be distinguished in most of the patients. When the size of the CVN was compared to that of the facial nerve on MRI, the diameter of the CVN was equal to or larger than the facial nerve in 2 cases and smaller in 3 cases (Fig. 3).

### Audiologic data and outcome of auditory rehabilitation

All of the patients demonstrated profound sensorineural hearing loss showing no response on auditory brainstem response test. Auditory performances evaluated at the time of initial visit were CAP 0 in all patients except for one who showed response to speech sounds (CAP 2) using hearing aids (Table 5).

**Table 5:** Auditory rehabilitation in Patients with CHARGE syndrome.

Patient No.	Age at CI (ABI)	Clinical diagnosis	CI site (ABI site)	CI Device	Pre-op CAP	Post-CI CAP	Post-ABI CAP	F/U after CI (after ABI)
1	3	Typical	R	Clarion 90K	0	6		2;8
2	20;8	Atypical	R	MedEl Pulsar	2	5		2;3
3	4;7	Atypical	R	Nucleus CI24R	0	4		3
4	2;5	Atypical	R	Nucleus CI24R	0	4		1;1
5	2;4	Atypical	R	Nucleus Freedom	0	4		2;4
6	1;2	Typical	R	Clarion 90K	0	2		1;6
7	2 (5;10)	Typical	R (R)	Clarion CII	0	0	1	4;7 (2)
8	4;7 (7;5)	Typical	L (L)	Clarion 90K	0	0	2	2;9 (0;1)
9	15**	Atypical	-		0	-	-	-

**Figure 4:**
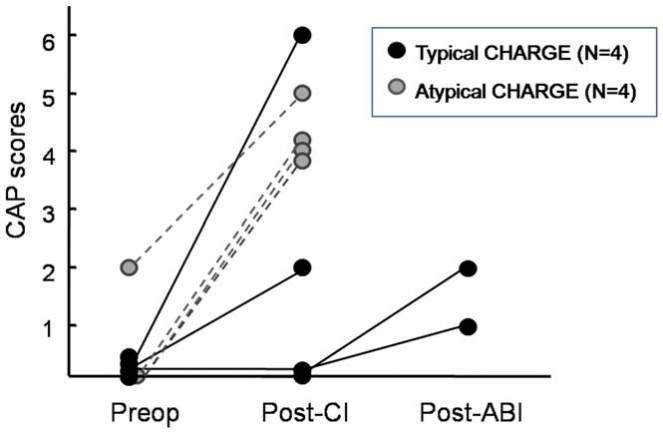
The improvement of auditory performance after cochlear implantation (CI) and auditory brainstem implantation (ABI) in patients with typical or atypical CHARGE syndrome.

CI was performed in 8 of 9 patients (Table 5). After at least 12 months of follow-up, 5 of 8 patients including one typical and four atypical CHARGE patients demonstrated significant improvement in auditory performances ranging from CAP 4 to 6 (Fig. 4). Among these patients showing good outcome, one patient aged 20 years (Patient 2) who exhibited residual hearing of CAP 2 preoperatively, improved to CAP 5 after 16 months following CI. Another patient with typical CHARGE syndrome (Patient 1) showed good outcome of CAP 6 after 2 years following CI, and the bisyllabic word identification improved from 0% to 70% without visual cues. One patient (Patient 6) showed suboptimal results after CI only reaching CAP 2 after 18 months postoperative to CI. In two patients, no sound detection could be achieved despite continued rehabilitation for more than 2 years following CI. Consequently, auditory brainstem implantation was performed in these two patients, following which improvements in auditory perception were perceived (Fig. 4).

No single factor seemed to be correlated with the outcome of auditory rehabilitation after CI in the patients. Although 3 of 4 patients diagnosed with typical CHARGE syndrome demonstrated poor outcome following CI (Patients 6, 7, and 8), Patient 1 was the best performer of all patients included in this study. Nevertheless, the three typical CHARGE patients (Patients 6, 7, and 8) all demonstrated coloboma, which may be related to poor prognosis after CI in CHARGE syndrome. The type of mutations of *CHD7* was not correlated with outcome. On radiologic evaluation, obliteration of BCNC was not a factor relevant to outcome, while MRI findings demonstrating no visible CVN or very thin CVN compared to the facial nerve at the cerebellopontine angle were more suggestive of poorer outcome. In addition, two patients who could not achieve any sound detection after CI both had severe combined disabilities such as blindness (Patient 7) and severe mental retardation (Patient 8), which seems to be associated with poor outcome after CI.

## Discussion

Five novel mutations and three previously reported mutations of the *CHD7* gene was identified in Korean patients with CHARGE syndrome presenting with profound hearing loss and typical inner ear anomalies. None of the mutations overlapped the previously reported mutations of the *CHD7* gene found in Korean patients [19]. The mutation rate was 89%, higher than previously reported despite inclusion of many patients with atypical CHARGE syndrome [8]. As Verloes [5] has emphasized, we believe that complete aplasia of the semicircular canals is a very specific finding that strongly suggests the presence of *CHD7* mutations even if other characteristic signs of CHARGE syndrome are missing. In comparison to the clinical features seen in *CHD7* mutation positive cohort in previous studies, the incidence of choanal atresia (13%) and coloboma (38%) were relatively lower in this report, which may be a finding more specific to the Korean population [6], [19].

Various types of mutations were encountered regardless of clinical diagnosis as typical or atypical CHARGE syndrome. Truncating mutations including nonsense and frameshift mutations were most commonly detected (63%), similar to the previous reports [8]. The patient who carried a missense mutation of the *CHD7* gene presented with the mildest symptoms demonstrating only mild developmental delay and mental retardation without any classical features of CHARGE syndrome other than hearing loss and semicircular canal aplasia. Three mutations identified in this study were previously reported; however, the phenotypes of patients having the same mutations differed considerably (Table S4) [20], [21]. No mutation was found in one patient diagnosed of typical CHARGE syndrome by direct sequencing of the 5′ UTR and coding region of the *CHD7* gene. There may be small intragenic deletions or mutations in the upstream regulatory region of the *CHD7* gene, or it is possible that a different gene responsible for other syndromes with similar phenotypes as CHARGE syndrome may be involved.

To date, 17 cases of molecularly confirmed familial CHARGE syndrome have been reported, which include seven sib-pairs, three monozygotic twin pairs, and seven two-generation families [6]. This study presents another familial case of CHARGE syndrome in which a sib-pair carried the same frameshift mutation causing truncation of the *CHD7* gene. Since no mutation was found in their parents, de novo mutation or germline mosaicism was suspected. The sister of the proband had hearing loss and semicircular canal aplasia only on one side unlike the proband who was diagnosed with typical CHARGE syndrome and bilateral ear abnormalities, demonstrating the intra-familial variability of CHARGE syndrome and also expanding the phenotypic spectrum of mildly affected patients.

Radiologic analysis of patients with typical and atypical CHARGE syndrome revealed that narrowing of the internal auditory canals and obliteration of the BCNC were also common findings in addition to aplasia of the semicircular canals typically seen in CHARGE syndrome. These findings have great clinical significance because of their correlation with CVN deficiency, which may lead to poor outcomes after CI [15], [22]. Indeed, CVN hypoplasia or aplasia was identified in all of our patients who underwent temporal MRI. Other than the anatomical factors, it is known that combined disabilities such as developmental delay, mental retardation, and blindness often seen in CHARGE syndrome act as hindering factors for proper auditory and speech developments [13].

Most of the previous studies dealing with the auditory outcome after CI or auditory brainstem implantation in CHARGE syndrome rely on clinical diagnosis only and did not perform genetic analysis of the *CHD7* gene [14], [16]. Since CHARGE syndrome is a clinically complex disorder, mutational analysis of the *CHD7* gene allows molecular confirmation of the diagnosis and also enables inclusion of mildly affected patients showing only few clinical features of CHARGE syndrome. Following CI, a wide range of auditory improvement was encountered from no sound perception to open-set speech discrimination without visual cues in our patients with CHARGE syndrome. The type of mutation of the *CHD7* gene did not demonstrate clear correlation with the prognosis after CI. Despite the presence of narrow internal auditory canals and obliterated BCNC, which may be considered as a contraindication to CI, the outcomes of CI were promising in majority of our patients [23]. In our opinion, CI should be recommended in CHARGE patients with profound hearing loss even if CVN deficiency or BCNC obliteration is present on imaging, especially when the size of the CVN is larger than or equal to that of the facial nerve on parasagittal view of MRI and severe mental retardation is not present.

In two patients with CHARGE syndrome who failed to perceive any sound stimulation with a CI, auditory brainstem implantation was performed and early results show increased attentiveness and responsiveness despite limited improvement in auditory perception. Auditory brainstem implantation in nontumor patients has shown promising outcomes recently, and we believe that auditory brainstem implantation is a viable option in patients with CHARGE syndrome in cases of failed stimulation after CI [24], [25]. Nevertheless, considering the difficulties in the pitch ranking process after auditory brainstem implantation in children that can be further complicated by the additional disabilities of CHARGE patients, long and strenuous rehabilitation may be warranted in these patients. Therefore, the parents must be counseled carefully and informed about the variability of outcome and the auditory rehabilitation process after CI or auditory brainstem implantation in patients with CHARGE syndrome.

In conclusion, 8 mutations of the *CHD7* gene including 5 novel mutations were identified in Korean patients with CHARGE syndrome showing semicircular canal aplasia and profound hearing loss, which will broaden the genotypic and phenotypic spectrum of CHARGE syndrome. Genetic analysis of the *CHD7* gene should be performed in cases with semicircular canal aplasia considering the high mutation rate even when the other typical features of CHARGE syndrome are not present. CI should be recommended in patients with CHARGE syndrome when the size of the CVN is larger than or equal to that of the facial nerve on MRI and mental retardation is not severe, since favorable outcome is expected in these cases. Auditory brainstem implantation may be considered in patients with CHARGE syndrome who have failed to benefit from CI.

## Supporting Information

Figure S1 **Multiple sequence alignment of the CHD7 protein orthologs.** CHD7 amino acid sequences of various vertebrate species are aligned using the Clustal W2 program (http://www.ebi.ac.uk/Tools/msa/clustalw2/). The region containing the novel missense mutation, p.R2065S (indicated as an asterisk), is highly conserved in vertebrates.
(TIF)

Figure S2 **Exon-trapping analysis of the novel splice site variation, c.5210+5G>C.** For the c.5210+5G>C variation, exons 22 and 23 of *CHD7* were introduced into the pSPL3 vector and analyzed by the *in vitro* splicing assay. For the wild type, normal 623-bp mRNA was identified. For the mutant type, 463-bp mRNA variant was seen together with the normal 623-bp mRNA (A). When sequencing analysis was performed, the normal 623-bp mRNA demonstrated both exons 22 and 23 between the pSPL3 exons (B), whereas the short 463-bp mRNA variant identified in the mutant type contained only exon 22 (C). SM, standard marker; WT, wild-type; MT, mutant type; N, negative.
(TIF)

Figure S3 **Exon-trapping analysis of splice site variation, c.5405-7G>A.** For the c.5405-7G>A variation, exon 26 of *CHD7* was introduced into the pSPL3 vector and analyzed by the *in vitro* splicing assay. For the wild type, normal 393-bp mRNA was identified, while the mutant type demonstrated only the 398-bp mRNA variant (A). Sequencing analysis of the wild type mRNA revealed exon 26 of *CHD7* between the pSPL3 exons (B), but the mutant mRNA variant sized 398-bp contained an additional 5-bp intronic sequence upstream of exon 26 (C). SM, standard marker; WT, wild-type; MT, mutant type; N, negative.
(TIF)

Table S1 **Primers and PCR conditions used for** ***CHD7*** **sequencing analysis.**
(DOC)

Table S2 **Splicing variants for** ***in vitro*** **splicing assay.** * The exon directly associated with the splice variation is presented in bold. Exon 22 of Patient 5 is closely located to exon 23 and was inserted into the pSPL3 vector together. Restriction enzyme recognition sites of the primer sequences are underlined.
(DOC)

Table S3 ***In silico*** **analysis through splice site prediction programs.** * Values in bold represent scores of the new splice site that is introduced by the intronic variation.
(DOC)

Table S4 **Comparison of the clinical features of patients with CHARGE syndrome who have the same mutations of the** ***CHD7*** **gene.** Of the three mutations identified in this study that had previously been reported, clinical features were provided for only two patients in the previous reports. TE: tracheoesophageal.
(DOC)
