# Supplementary figures and images for: CHD7 Mutational Analysis and Clinical Considerations for Auditory Rehabilitation in Deaf Patients with CHARGE Syndrome
Source: PLoS One. 2011 Sep 13;6(9):e24511. doi: 10.1371/journal.pone.0024511 (PMC3172230; doi:10.1371/journal.pone.0024511)

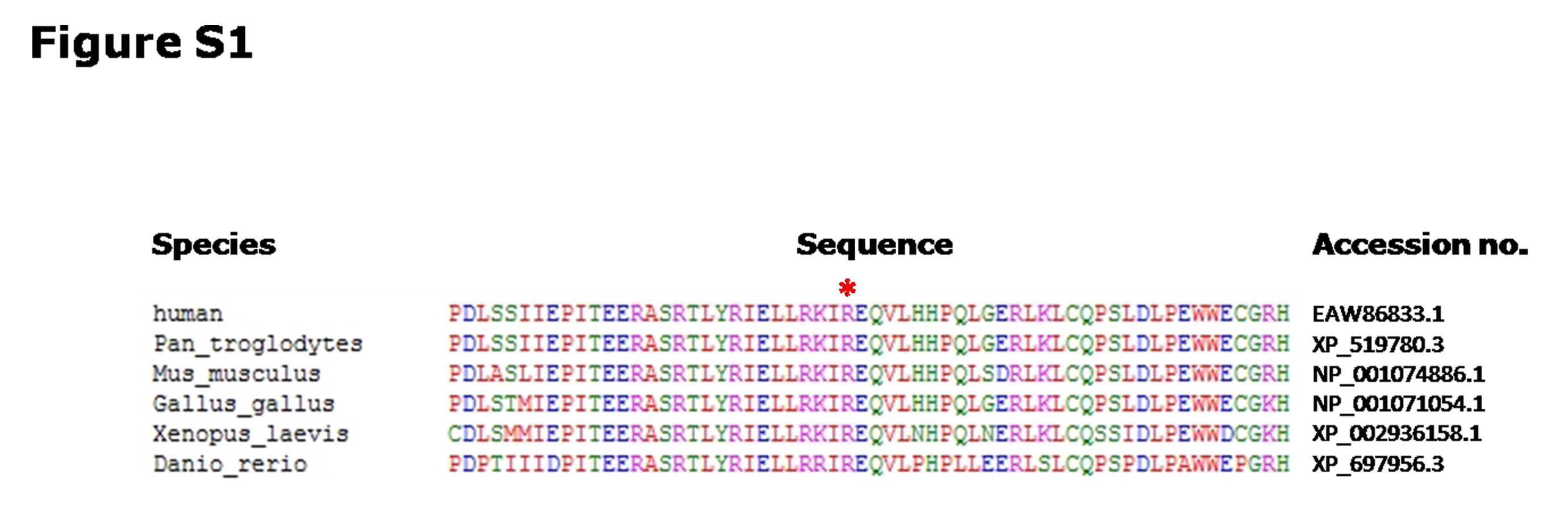

Supplement: Figure S1 — Multiple sequence alignment of the CHD7 protein orthologs. CHD7 amino acid sequences of various vertebrate species are aligned using the Clustal W2 program (http://www.ebi.ac.uk/Tools/msa/clustalw2/). The region containing the novel missense mutation, p.R2065S (indicated as an asterisk), is highly conserved in vertebrates. (TIF) [file pone.0024511.s001.tif]

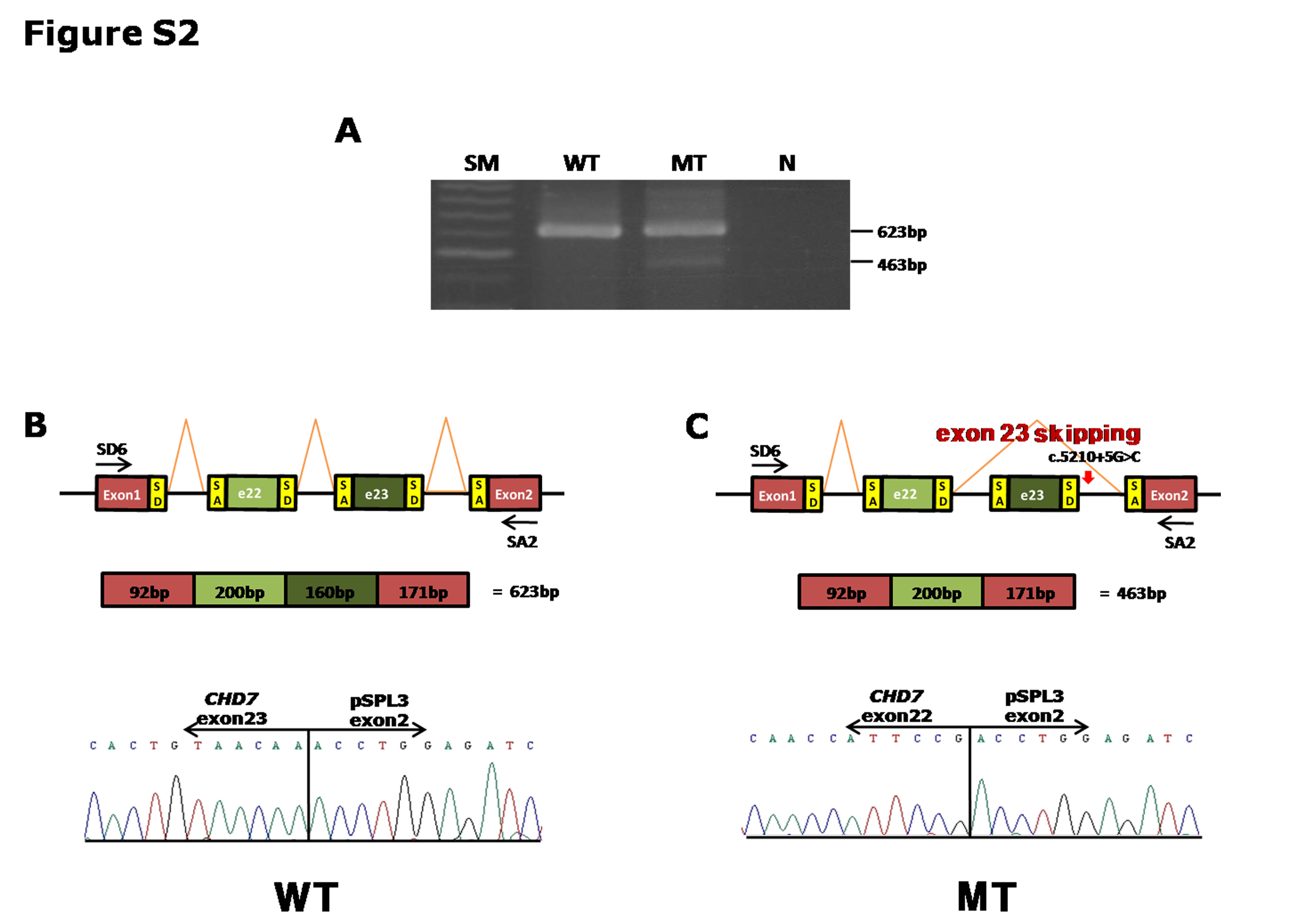

Supplement: Figure S2 — Exon-trapping analysis of the novel splice site variation, c.5210+5G>C. For the c.5210+5G>C variation, exons 22 and 23 of CHD7 were introduced into the pSPL3 vector and analyzed by the in vitro splicing assay. For the wild type, normal 623-bp mRNA was identified. For the mutant type, 463-bp mRNA variant was seen together with the normal 623-bp mRNA (A). When sequencing analysis was performed, the normal 623-bp mRNA demonstrated both exons 22 and 23 between the pSPL3 exons (B), whereas the short 463-bp mRNA variant identified in the mutant type contained only exon 22 (C). SM, standard marker; WT, wild-type; MT, mutant type; N, negative. (TIF) [file pone.0024511.s002.tif]

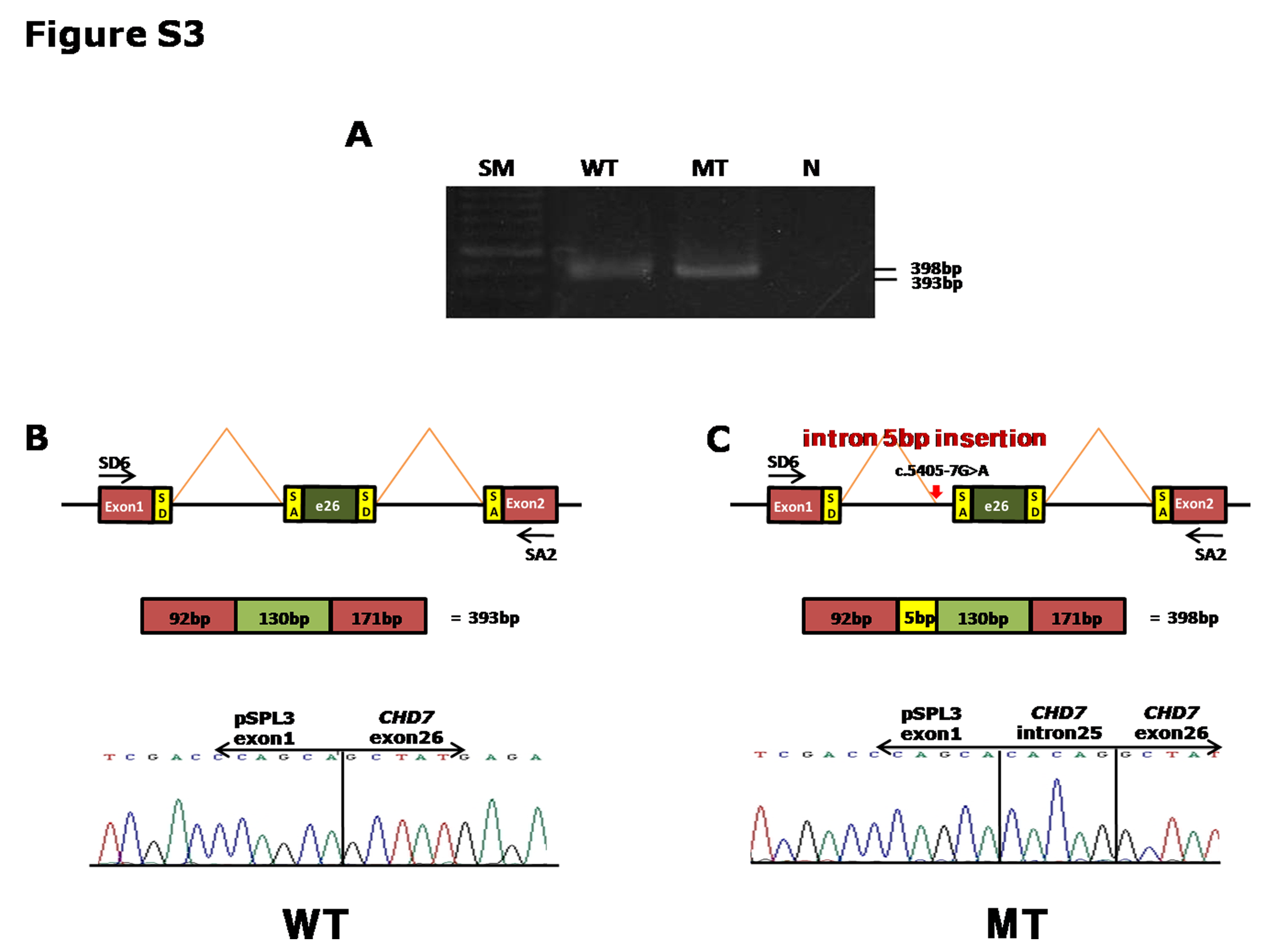

Supplement: Figure S3 — Exon-trapping analysis of splice site variation, c.5405-7G>A. For the c.5405-7G>A variation, exon 26 of CHD7 was introduced into the pSPL3 vector and analyzed by the in vitro splicing assay. For the wild type, normal 393-bp mRNA was identified, while the mutant type demonstrated only the 398-bp mRNA variant (A). Sequencing analysis of the wild type mRNA revealed exon 26 of CHD7 between the pSPL3 exons (B), but the mutant mRNA variant sized 398-bp contained an additional 5-bp intronic sequence upstream of exon 26 (C). SM, standard marker; WT, wild-type; MT, mutant type; N, negative. (TIF) [file pone.0024511.s003.tif]
