# Supplementary material for: CHD7 Mutational Analysis and Clinical Considerations for Auditory Rehabilitation in Deaf Patients with CHARGE Syndrome
Source: PLoS One. 2011 Sep 13;6(9):e24511. doi: 10.1371/journal.pone.0024511 (PMC3172230; doi:10.1371/journal.pone.0024511)
Supplement: Table S1 — Primers and PCR conditions used for CHD7 sequencing analysis. (DOC) [file pone.0024511.s004.doc]

**Table S1.** Primers and PCR conditions used for *CHD7* sequencing analysis.

| **Primer name** | **Sequence (5'-3')** | **Target region** | **Product size**  **(base pairs, bp)** | **Annealing temperature (℃)** |
| --- | --- | --- | --- | --- |
| CHD7_e1F | cggccgaattcttgcataaa | exon 1 | 602 | 57 |
| CHD7_e1R | ctcctcgctcccaactccta |
| CHD7_e2-1F | tggggaccaaattcagatgt | exon 2 | 669 | 57 |
| CHD7_e2-1R | attctgcatgccagggtaga |
| CHD7_e2-2F | ccgaacagaatgatgagcaac | exon 2 | 642 | 57 |
| CHD7_e2-2R | gactgtctggctccgagaac |
| CHD7_e2-3F | tcaccacccctctactgctc | exon 2 | 630 | 57 |
| CHD7_e2-3R | gccgaggacatgcctataaa |
| CHD7_e2-4F | taatccagcaggcactcctc | exon 2 | 460 | 57 |
| CHD7_e2-4R | tacctgatgcacaggcactt |
| CHD7_e3F | tttttggatgagagaagctgat | exon 3 | 681 | 53 |
| CHD7_e3R | acccctcatttcataggctgt |
| CHD7_e4F | ttgccaggaaaacttaattgg | exon 4 | 361 | 57 |
| CHD7_e4R | gctaaagttacccatgccaaa |
| CHD7_e5F | aaaattcatccttccactctgc | exon 5 | 636 | 57 |
| CHD7_e5R | gctgaaagtccaatgcttcc |
| CHD7_e6F | acctaggctggtccacgtaa | exon 6 | 479 | 57 |
| CHD7_e6R | ctggggtcaaatatcccaaag |
| CHD7_e7F | tagaagctctcgcacgttga | exon 7 | 401 | 57 |
| CHD7_e7R | ccaggccatgatgactaaaga |
| CHD7_e8F | gagctggaggcttaggacag | exon 8 | 452 | 57 |
| CHD7_e8R | caagttgacagcaccaaatga |
| CHD7_e9F | tcacagtaaaaggtttcacacg | exon 9 | 426 | 57 |
| CHD7_e9R | aaggctctgaccaagaccag |
| CHD7_e10-11F | tgtatgtggtcaaatgaatccaa | exon 10,11 | 469 | 57 |
| CHD7_e10-11R | tcaataactaaaggaaaggaactacag |
| CHD7_e12F | tgcatttgtgggtacaatgg | exon 12 | 428 | 57 |
| CHD7_e12R | ccttcccaagtcaccaagac |
| CHD7_e13F | gatgcatgtgctctgttagga | exon 13 | 492 | 57 |
| CHD7_e13R | tctgagcaacgggactatca |
| CHD7_e14F | tgtccatggatctgtctacctg | exon 14 | 489 | 57 |
| CHD7_e14R | tgatgacctggaaagtccaa |
| CHD7_e15F | caaccttcatgcactgctct | exon 15 | 494 | 57 |
| CHD7_e15R | aagggatttctggtgcttca |
| CHD7_e16F | aatgcctcaatgtgttgtgc | exon 16 | 486 | 57 |
| CHD7_e16R | gacccctgggaaggtaattt |
| CHD7_e17F | cgccaataaaccctatttgct | exon 17 | 457 | 57 |
| CHD7_e17R | ctgagtgacgacgcaacatt |
| CHD7_e18F | acattggaatgagggtttcag | exon 18 | 550 | 57 |
| CHD7_e18R | gccccttttcgtagaagatcc |
| CHD7_e19F | gcctttgattatttttcgacctc | exon 19 | 478 | 57 |
| CHD7_e19R | aggggttccacagggagtta |
| CHD7_e20-21F | tgtctggcataagtggagga | exon 20,21 | 596 | 57 |
| CHD7_e20-21R | ttctctaccagttgggaattcta |
| CHD7_e22-23F | cacccagtgtgaattgttgc | exon 22,23 | 759 | 57 |
| CHD7_e22-23R | ctgcatctgcagacgtgaag |
| CHD7_e24F | aggcaatggtagggtacgtg | exon 24 | 585 | 57 |
| CHD7_e24R | acagccttttcctgattctca |
| CHD7_e25F | tggcagagggctactgactc | exon 25 | 572 | 57 |
| CHD7_e25R | cgctggaagcaaagtttagg |
| CHD7_e26F | ccaggcatcttgcagttattc | exon 26 | 466 | 57 |
| CHD7_e26R | gaaccctgccaatagatgtga |
| CHD7_e27-28F | atgtcatttcccgcaatctcc | exon 27,28 | 554 | 57 |
| CHD7_e27-28R | accacgtgaacaatgactgc |
| CHD7_e29F | taaaaatgagggcactgagatg | exon 29 | 538 | 57 |
| CHD7_e29R | cccccagactttatacatggtag |
| CHD7_e30F | ggtagtgaccaccaaagaaagg | exon 30 | 532 | 57 |
| CHD7_e30R | ctctgtgatcggctcaattatg |
| CHD7_e31-1F | gccatgtgtaggcgagtatgt | exon 31 | 642 | 57 |
| CHD7_e31-1R | cctcttctttgccctcacatt |
| CHD7_e31-2F | caaaacagaggggcaggtaata | exon 31 | 552 | 57 |
| CHD7_e31-2R | ataaggtgcgagagcaaaacac |
| CHD7_e32F | gacaacagtgcccaatacca | exon 32 | 578 | 57 |
| CHD7_e32R | atggagccaaagatcaatcc |
| CHD7_e33F | ctaggaccttctgccagagc | exon 33 | 531 | 57 |
| CHD7_e33R | tttctaagcaaggccagtgaa |
| CHD7_e34F | ctcagctctgtgcaccagtcat | exon 34 | 689 | 57 |
| CHD7_e34R | ggaagctggctttcatacaatg |
| CHD7_e35F | ccagcctcattttctgactttt | exon 35 | 544 | 57 |
| CHD7_e35R | aacaacaaagacctgggaaatg |
| CHD7_e36F | ccttaatggacgggtaaaatagg | exon 36 | 586 | 57 |
| CHD7_e36R | cacctgggtagatccttcattg |
| CHD7_e37F | tttcttagcccagaaggaagtg | exon 37 | 539 | 57 |
| CHD7_e37R | cccctggagaagtctacctctaa |
| CHD7_e38-1F | tgagctgttaggagggaagaac | exon 38 | 532 | 57 |
| CHD7_e38-1R | tttccagtagcagctgacagag |
| CHD7_e38-2F | gaatctccagaatctccagtcg | exon 38 | 670 | 57 |
| CHD7_e38-2R | gtctagctcttcaccctgtgct |
| CHD7_e38-3F | tccatgtttctacctccaggac | exon 38 | 630 | 57 |
| CHD7_e38-3R | tgagcagcactttcttccatta |
