# Supplementary material for: CHD7 Mutational Analysis and Clinical Considerations for Auditory Rehabilitation in Deaf Patients with CHARGE Syndrome
Source: PLoS One. 2011 Sep 13;6(9):e24511. doi: 10.1371/journal.pone.0024511 (PMC3172230; doi:10.1371/journal.pone.0024511)
Supplement: Table S2 — Splicing variants for in vitro splicing assay. * The exon directly associated with the splice variation is presented in bold. Exon 22 of Patient 5 is closely located to exon 23 and was inserted into the pSPL3 vector together. Restriction enzyme recognition sites of the primer sequences are underlined. (DOC) [file pone.0024511.s005.doc]

**Table S2.** Splicing variants for *in vitro* splicing assay.

| Variation | Subject | Type of  Splice-site | Insertion fragment to mini-gene system* | | |
| --- | --- | --- | --- | --- | --- |
|  |  |  | Exon | Forward primer | Reverse primer |
| c.5210+5G>C | Patient 5 | Donor | 22, **23** | 5’-cggaattcggcagcttactgtataaaggagtg-3’ | 5’-cgggatccacgttgtgttgtgtatcttaccatg-3’ |
| c.5405-7G>A | Patient 7 | Acceptor | **26** | 5’-cggaattcgtgtttgtggtaattctgataa-3’ | 5’-cgggatccgggaaatgacatttggaaaag-3’ |

* The exon directly associated with the splice variation is presented in bold. Exon 22 of patient 5 is closely located to exon 23 and was inserted into the pSPL3 vector together.

Restriction enzyme recognition sites of the primer sequences are underlined.
