# Supplementary material for: CHD7 Mutational Analysis and Clinical Considerations for Auditory Rehabilitation in Deaf Patients with CHARGE Syndrome
Source: PLoS One. 2011 Sep 13;6(9):e24511. doi: 10.1371/journal.pone.0024511 (PMC3172230; doi:10.1371/journal.pone.0024511)
Supplement: Table S3 — In silico analysis through splice site prediction programs. * Values in bold represent scores of the new splice site that is introduced by the intronic variation. (DOC) [file pone.0024511.s006.doc]

| Variation | Prediction program | Normal score | Mutant score | Variation (%) |
| --- | --- | --- | --- | --- |
| c.5210+5G>C | FruitFly | 0.57 | Not found |  |
|  | NetGene2 | 0.82 | 0.24 | - 70.7 |
|  | Human Splicing Finder | 80.62 | 68.6 | - 14.9 |
| c.5405-7G>A | FruitFly | Not found | Not found |  |
|  | NetGene2 | **Not found** | **0.34** |  |
|  | Human Splicing Finder | **46.02** | **74.95** | **+ 62.91** |

**Table S3.** *In silico* analysis through splice site prediction programs.

* Values in bold represent scores of the new splice site that is introduced by the intronic variation.
