# Supplementary material for: CHD7 Mutational Analysis and Clinical Considerations for Auditory Rehabilitation in Deaf Patients with CHARGE Syndrome
Source: PLoS One. 2011 Sep 13;6(9):e24511. doi: 10.1371/journal.pone.0024511 (PMC3172230; doi:10.1371/journal.pone.0024511)
Supplement: Table S4 — Comparison of the clinical features of patients with CHARGE syndrome who have the same mutations of the CHD7 gene. Of the three mutations identified in this study that had previously been reported, clinical features were provided for only two patients in the previous reports. TE: tracheoesophageal. (DOC) [file pone.0024511.s007.doc]

| Clinical Features | c.5405-7G>A | |  | p.Q489X | |
| --- | --- | --- | --- | --- | --- |
|  | Patient 7 | Arakami (23) |  | Patient 9 | Jongman (22) |
| **Major** |  |  |  |  |  |
| Coloboma | + | + |  | - | + |
| Choanal atresia | - | - |  | - | - |
| Semicircular canal aplasia | + | ? |  | + | ? |
| **Minor** |  |  |  |  |  |
| Rhomencephalic dysfunction | +  (profound hearing loss) | +  (moderate hearing loss) |  | + | ? |
| Hypothalamohypophyseal dysfunction | + | + |  | + | - |
| Abnormal middle/external ear | - | +  (external ear) |  | + | + |
| Malformation of mediastinal organs | +  (Heart defect) | +  (laryngomalacia/TE Fistula) |  | - | +  (Heart defect) |
| Mental retardation | + | ? |  | - | + |
| **Others** |  |  |  |  |  |
| Urogenital anomalies | - | + |  | + | - |
| Cleft palate/lip | - | + |  | - | + |
| Limb abnormalities | - | ? |  | + | - |
| Facial dysmorphia | - | ? |  | - | ? |

**Table S4.** Comparison of the clinical features of patients with CHARGE syndrome who have the same mutations of the *CHD7* gene.

Of the three mutations identified in this study that have previously been reported, clinical features were provided for only two patients.

TE: tracheoesophageal.
